# Shear Wave Tensiometry in the Evaluation of Achilles Tendon Loading: A Cross‐Sectional Study on Conservatively Treated Tendons After Rupture

**DOI:** 10.1002/jfa2.70047

**Published:** 2025-04-27

**Authors:** Alessandro Schneebeli, Giuseppe Filardo, Enrique Testa, Martin Riegger, Deborah Falla, Alessandro Sangiorgio, Corrado Cescon, Emiliano Soldini, Marco Barbero

**Affiliations:** ^1^ Centre of Precision Rehabilitation for Spinal Pain (CPR Spine) School of Sport, Exercise and Rehabilitation Sciences College of Life and Environmental Sciences University of Birmingham Birmingham UK; ^2^ Rehabilitation Research Laboratory 2rLab Department of Business Economics Health and Social Care University of Applied Sciences and Arts of Southern Switzerland Manno Switzerland; ^3^ Service of Orthopaedics and Traumatology Department of Surgery EOC Lugano Switzerland; ^4^ Faculty of Biomedical Sciences Università Della Svizzera Italiana Lugano Switzerland; ^5^ Service of Orthopaedics and Traumatology Department of Surgery EOC Bellinzona Switzerland; ^6^ Clinics of Rehabilitation Ente Ospedaliero Cantonale (CREOC) Novaggio/Faido Switzerland; ^7^ Competence Centre for Healthcare Practices and Policies Department of Business Economics, Health, and Social Care University of Applied Sciences and Arts of Southern Switzerland Manno Switzerland

**Keywords:** achilles tendon rupture, conservative treatment, shear wave tensiometry, tendon loading

## Abstract

**Purpose:**

The aim of this study was to quantify differences in the shear wave speed (SWS) between a conservatively treated Achilles tendon (AT) after rupture and the unaffected contralateral tendon.

**Methods:**

Twenty‐nine participants who received conservative treatment following Achilles tendon rupture (ATR) were enrolled. Measurements were taken during a single follow‐up visit, which occurred between 1 and 7 years after the rupture. Tendon load was assessed using a shear wave tensiometer comprising a set of four accelerometers attached to the tendon. Stiffness, thickness, and cross‐sectional area (CSA) were also assessed using MyotonPRO and ultrasound imaging.

**Results:**

No significant differences in SWS were found between the affected AT and the unaffected side when analyzing the entire group (*p* > 0.05). However, significant differences between sides were observed at 3.5 Nm and 7 Nm (*p* = 0.001 and *p* = 0.020) for participants that experienced a lesion of the mid tendon. Higher plantar flexor strength was found for the unaffected side (320 ± 99.5 Nm) compared to the affected side (261 ± 80 Nm; *p* = 0.001). Thickness and CSA in the proximal and distal part of the tendon were significantly higher in the affected tendon compared to the unaffected side (*p* < 0.001).

**Conclusion:**

There is no difference in SWS values between the affected and the unaffected AT in the longer term after the rupture. However, differences in SWS were detected at specific contraction levels in participants with a mid‐tendon lesion. Moreover, tendon thickness and the cross‐sectional area, as well as plantar flexor strength, remain different between the affected and the unaffected AT.

## 1 Introduction

The most suitable treatment for Achilles tendon rupture (ATR) is still not defined and remains controversially discussed in the literature. A mounting body of evidence indicates comparable long‐term patient‐reported outcomes for both surgical and conservative treatment [1, 2, 3]. These findings were confirmed by Ochen et al. [4] in their comprehensive systematic review, which demonstrated comparable patient‐reported outcome measures for both treatments. However, their analysis revealed a higher risk of re‐rupture associated with conservative treatment and a greater incidence of postoperative complications in surgically treated Achilles tendons (AT).

A recent large clinical trial involving a cohort of over 500 patients diagnosed with ATR aimed at discerning differences among open repair surgery, minimally invasive surgery, and conservative treatment concerning patient‐reported outcomes and the incidence of tendon re‐rupture [3]. The findings revealed that 12 months post‐ATR, all three approaches exhibited similar results in the assessed outcomes. The conservative treatment group experienced a lower occurrence of nerve injuries. However, the conservative treatment group exhibited a slightly higher re‐rupture rate (6.2%) than the surgical groups (0.6%).

The mechanical properties of the AT post‐surgery have been assessed using various technologies at different stages of the healing process [5, 6, 7, 8, 9, 10, 11, 12]. However, the mechanical properties of AT after conservative treatment have not been well documented in the literature. The few studies that have explored differences between the affected tendon and the unaffected side have observed no differences in the mechanical properties of the tendons after 1 year [13] or even a higher tendon stiffness in the affected compared to the contralateral tendon [14].

Shear wave tensiometry is a novel technology that uses accelerometers to track shear wave speed propagation along the tendon [15, 16, 17]. Tendon loading is calculated based on the concept of axial stress within the tissue which affects the squared tendon wave speed. This technology has been applied in different settings, evaluating healthy participants in order to define AT loading during locomotion [18, 19] and has been shown to be able to detect differences in tendon mechanical properties between the operated and healthy AT in patients with ATR after surgery [20] providing a better understanding of the mechanical behavior of tendons after surgery. Evaluating the mechanical properties of tendons undergoing conservative treatment after rupture could help, in future studies, to determine whether there is an association between altered mechanical properties and the risk of AT re‐rupture.

The aim of this study was to evaluate differences in shear wave speed between conservatively treated ATs after rupture and the unaffected contralateral tendons. Additionally, morphological properties, thickness, and cross‐sectional area (CSA) of the tendons were measured using ultrasound, and tendon stiffness was measured using myotonometry.

## 2 Materials and Methods

The study was carried out at the Orthopedic Department of the Ente Ospedaliero Cantonale (Lugano and Bellinzona, Switzerland). Approval for the study was obtained from the Ethics Committee of Canton Ticino, Switzerland (ID: 2022‐01707/CE4192), and participants provided written informed consent. Reporting of the study followed the STROBE guidelines for observational studies [21].

### 2.1 Participant Selection

Participants who underwent conservative treatment following full‐thickness mid Achilles tendon rupture or partial rupture of the myotendinous junction between 10 and 90 months prior to selection were identified through the hospitals' database and those who had been diagnosed with ATR by an orthopedic surgeon in the hospital’s emergency department were contacted. We included all patients aged 18 or more without surgical treatment for ATR. Exclusions criteria were

— arthrodesis of one of the upper ankle joints
— history of contralateral AT disease
— connective tissue or metabolic diseases
— systemic inflammatory disorders
— rheumatoid arthritis
— spondyloarthropathy
— diabetes
— current use of systemic antibiotics or steroids

All recruited participants underwent measurements of mechanical and morphological properties of the AT, as well as clinical assessments.

### 2.2 Clinical Outcomes

Participants completed health‐related physical activity questionnaires and the Achilles tendon total rupture score questionnaire (ATRS) [22]. The Tegner activity level scale was used to evaluate the level of physical activity before and after tendon rupture [23]. Ankle dorsiflexion range of motion, calf circumference, and maximal voluntary contraction (MVC) were also measured. Information about the location of the rupture was recorded. The injury location was then confirmed by ultrasound examination. The criteria for identifying a mid‐tendon lesion or a myotendinous junction lesion included the presence of tendon thickening in the mid‐tendon area, disorganization of tendon fibers, and hypoechoic areas in the affected region. Ultrasound screening, as well as the collection of the data, was performed by a researcher with 10 years of experience in tendon evaluation using ultrasound and supervised by an orthopedic surgeon. All clinical evaluation data were collected during a single visit.

### 2.3 Shear Wave Tensiometry

Tendon loading was evaluated using a shear wave tensiometer, which comprised four accelerometers (Analog Devices, ADXL202JE) arranged sequentially and affixed to the skin behind the tendon using an adhesive tape. These accelerometers were evenly spaced at 10 mm intervals. To ensure correct placement on the free section of the AT (3–6 cm from the calcaneal insertion) and not on the musculotendinous junction, ultrasound imaging was employed for guidance. The minimal impulse required to initiate wave propagation along the tendon was manually administered with a reflex hammer. A series of five repeated measurements (one per second) was obtained for each contraction level, for both the left and right AT of each participant. The mean of these five measurements was then used to calculate the tendon wave speed. The reliability of this technology was assessed in a prior study, revealing good to excellent reliability (intraclass correlation coefficient 0.75–0.96) [12].

**FIGURE 1:**
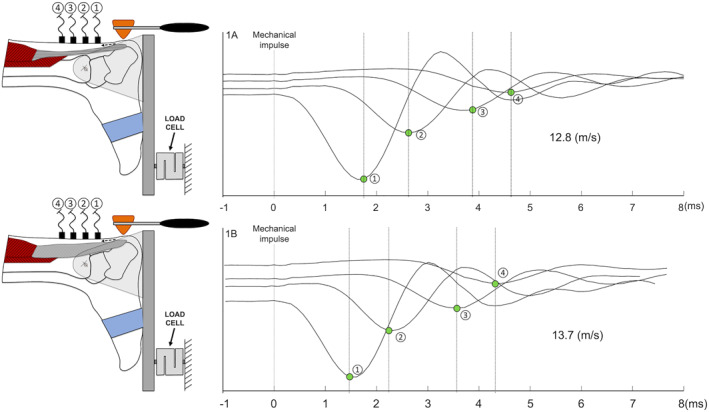
Schematic of the shear wave tensiometer. The impulse was provided at 2 cm from the calcaneal insertion. The 4 accelerometers (1–4) were attached to the skin in series with a 10‐mm interaccelerometer distance. Shear wave speed signals at 0 Nm. The propagation velocity of the wave was calculated based on the delay between the waves passing from the accelerometers. (1A) Represents a shear wave speed signals at 0 Nm for the unaffected tendon, whereas (1B) represents a shear wave speed signals at 0 Nm for the affected tendon.

The signals were acquired using a Sessantaquattro device (OTBioelettronica, Turin, Italy) with a sampling frequency of 10 kHz. The analysis of signals relied on the time delay between waves generated by the accelerometers and the propagation of the mechanical wave along the tendon (Figure 1). The technique for estimating the propagation velocity employed cross‐correlation functions between pairs of signals [24, 25]. Each impulse corresponded to a propagation speed value in meters per second (m/s).

During these measurements, participants assumed a prone position with their feet securely fastened in a dynamometer at a 0‐degree angle of plantar flexion. Ten different isometric contractions, ranging from 0 to 35 Nm in intensity, were performed. Ankle torque was assessed using a dynamometer linked to a force sensor (Mod.TF2/S; CCT Transducers, Turin, Italy), which functioned within a 0‐ to 100‐N range. A MISO‐II device (OT Bioelettronica, Turin, Italy) was employed to provide real‐time visual feedback of the force signal to the participants. The participants were instructed to exert force on the board as if they were trying to achieve a tiptoe position. The investigator monitored their performance to ensure that the heel remained in contact with the dynamometer, and two belts were used to secure a firm fixation of the foot and ankle.

### 2.4 Myotonometry

Tendon mechanical properties were also measured using a commercially available device called MyotonPRO (Muomeetria, Tallinn, Estonia). This device detects viscoelastic properties of different tissues and was applied following a standardized protocol [26]. Both the affected and unaffected AT were measured at two distinct locations along the free tendon: a distal region located 3 cm from the calcaneal insertion and a proximal region located 6 cm from the calcaneal insertion. Participants’ feet were hanging freely over the edge of the examination bed during the measurements. Myotonometry was used to evaluate the state of the tendon at rest. This, together with the assessment under load performed with shear wave tensiometry, enabled a better characterization of AT behavior.

### 2.5 Ultrasound Imaging

**FIGURE 2:**
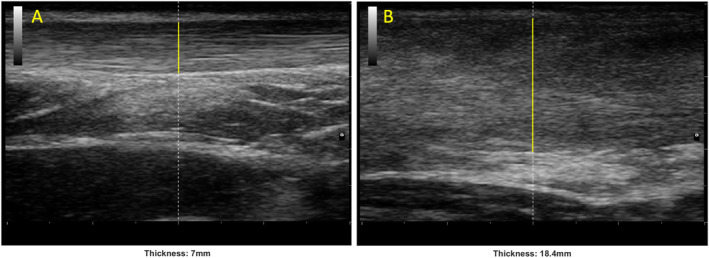
Ultrasound images representing the thickness of the Achilles tendon in a longitudinal scan. (A) Unaffected side and (B) affected side.

Ultrasound images were acquired to measure the thickness and CSA of both the affected and unaffected AT by the same experienced operator that performed the screening. The images were obtained using an ultrasound machine (MyLab25 gold, Esaote, Genoa, Italy) with a 13‐ to 4‐MHz linear array (LA523). The distances for the measurements were determined using standardized anatomical landmarks. The more proximal part of the AT insertion at the calcaneus bone was visualized and marked on the skin; a mark on the skin was then made every 1 cm from the reference point. Thickness images were obtained through a longitudinal scan, covering the range from 2 to 7 cm from the calcaneal insertion (Figure 2). In contrast, CSA images were captured through a transverse scan conducted at the 3 and 6 cm positions from the calcaneal insertion. The operator followed a standardized protocol that considered probe alignment and probe pressure on the skin. Moreover, to minimize variability and to ensure reproducibility of the measurements ultrasound settings were the same for each participant. The reliability of ultrasound measures of morphological parameters of the AT have been reported as good to excellent [27].

### 2.6 Study Size

Sample size was calculated based on the mean and standard deviation of the paired differences between the affected and unaffected AT found in a previous study [20] using Stata (version 17). A paired *t*‐test was employed to determine the minimum sample size required to achieve a statistical power of 90% at a significance level of 5% for a two‐tailed hypothesis test. Based on these parameters, a minimum sample size of 30 participants was required.

### 2.7 Statistical Analysis

The nonparametric, independent samples, Mann–Whitney test was used to assess differences in shear wave speed between participants with a different injury location (mid tendon or myotendinous junction).

The nonparametric Friedman test was used to assess differences in AT wave speed and other outcomes between the affected and the unaffected sides. Post hoc pairwise comparisons with Bonferroni correction were performed, and significance was set at *α* = 0.05. Linear regression models were used to test the association between wave speed and ankle joint torque. For each tendon, a linear regression model was used to test the relation between squared wave speed and ankle joint torque. The model's goodness of fit was assessed using the coefficient of determination (R2). Wave speed values were presented as median and interquartile range (IQR). The statistical analysis was conducted using SPSS (version 28).

## 3 Results

Thirty‐two participants were approached and screened resulting in three participants being excluded (two bilateral ruptures, one with pain during the isometric contraction). Thus, 29 participants (three women) were recruited for the study. Their mean (± SD) age was 54.8 (± 9.0) years and the mean time from rupture was 36.0 (± 24.2) months (range 10–90 months). Fifteen participants experienced a complete rupture of the mid tendon (MID), whereas 14 experienced a partial rupture at the level of the musculotendinous junction (MTJ). The demographics of both groups are presented in Table 1. No between group differences were found for age (*p* = 0.387), height (*p* = 0.141), weight (*p* = 0.113), BMI (*p* = 0.245), or time from rupture (*p* = 0.309).

**TABLE 1:** Demographics and clinical features of the enrolled participants.

Participants (N)	Age (y) (SD)	Hight (cm) (SD)	Weight (kg) (SD)	BMI (SD)	Time from rupture (months min–max)	ATRS (min–max)	Tegner (%)
Total (31)	54.8 (9)	176.6 (7.3)	82.1 (16.2)	26.2 (4.0)	36 (10–90)	85.5 (35–100)	72%
MID group (15)	53.5 (8)	178.5 (6.8)	84.9 (14.2)	26.6 (4.8)	34 (11–80)	83 (35–100)	69%
MTJ group (14)	56.4 (10)	174.5 (7.5)	79.3 (10.7)	25.8 (2.6)	38 (10–90)	88 (66–100)	74%
Between group difference	*p* = 0.387	*p* = 0.141	*p* = 0.113	*p* = 0.245	*p* = 0.309	*p* = 0.216	*p* = 0.121

### 3.1 Clinical Outcomes

The Tegner activity level scale indicated that 72% of participants were able to resume a similar level of sports activity as before the operation. The ATRS at follow‐up was 86/100 ± 16.1 ranging from 35 to 100. Clinical features of both groups are presented in Table 1; no between group differences were found for the Tegner activity scale (*p* = 0.121) or ATRS (*p* = 0.216). The average ankle dorsiflexion range of motion was not different between the affected and unaffected sides (*p* = 0.127). The mean calf circumference was significantly higher for the leg on the unaffected side compared to the affected side (*p* = 0.001) measuring 38 ± 3 cm versus 37 ± 3.3 cm, respectively. MVC was different between the affected and the unaffected sides, with a higher torque generated on the unaffected side, 320 ± 99.5 Nm, compared to the affected side, 261 ± 80 Nm (*p* = 0.001).

### 3.2 Shear Wave Tensiometry

**FIGURE 3:**
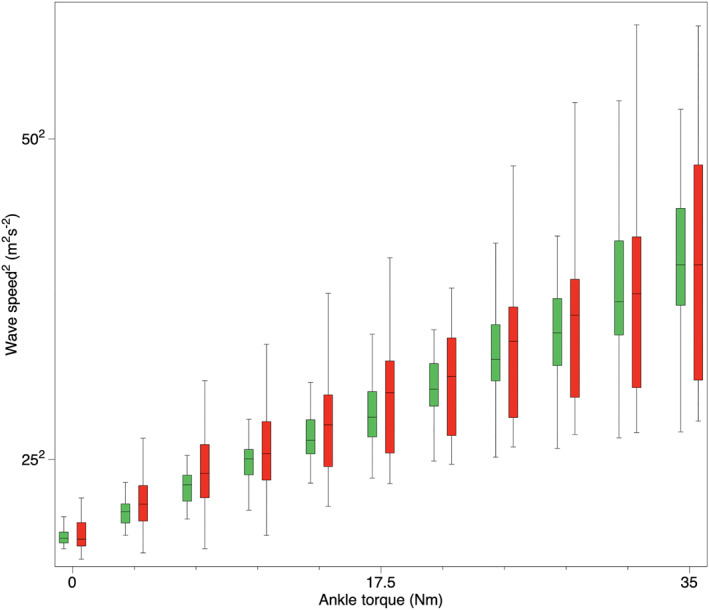
Box plot showing the median (interquartile range) squared shear wave speed of the entire sample group at different ankle torques. Green boxes and red boxes represent the unaffected and the affected sides, respectively.

Table 2 and Figure 3 present the shear wave speed values of the affected and unaffected AT measured at various contraction intensities. The Friedman test revealed no significant difference between sides for each of the contraction levels analyzed (*p* > 0.059) when the entire sample was evaluated.

**TABLE 2:** Shear wave speed differences between the affected and the unaffected tendons in the entire sample and divided in the two groups at different ankle torques. Boldface indicates significance (*p* < 0.05).

		SWS median (IQR) (m/s)					
		Total	*p*	MTJ	*p*	MID	*p*
0 Nm	H	12.86 (2.57)	0.257	13.39 (2.21)	0.782	12.18 (2.21)	0.071
	RUP	13.01 (5.29)		12.28 (3.37)		15.87 (6.99)	
3.5 Nm	H	17.89 (3.22)	0.059	19.13 (1.56)	0.405	16.58 (3.59)	**0.001**
	RUP	19.17 (6.03)		17.94 (5.72)		22.20 (5.23)	
7 Nm	H	21.86 (3.72)	0.059	22.82 (1.95)	0.782	19.67 (3.39)	**0.020**
	RUP	23.38 (6.67)		21.86 (6.23)		25.46 (6.94)	
10.5 Nm	H	25.08 (3.18)	0.450	25.81 (2.08)	0.782	23.68 (3.92)	0.197
	RUP	26.06 (6.87)		25.44 (6.23)		26.94 (8.93)	
14 Nm	H	27.17 (3.65)	0.450	29.25 (3.55)	0.782	25.76 (4.07)	0.197
	RUP	28.95 (7.51)		27.96 (7.00)		30.34 (10.37)	
17.5 Nm	H	29.54 (4.59)	0.257	31.09 (4.93)	0.782	29.01 (4.91)	0.197
	RUP	32.10 (8.83)		29.80 (8.73)		33.50 (7.46)	
21 Nm	H	32.19 (3.93)	0.705	32.98 (5.04)	0.405	31.03 (4.43)	0.197
	RUP	33.57 (9.04)		30.93 (9.39)		34.68 (8.28)	
24.5 Nm	H	34.79 (4.94)	0.705	36.55 (6.67)	0.052	33.72 (5.42)	0.197
	RUP	36.35 (9.65)		33.64 (9.98)		37.72 (9.14)	
28 Nm	H	36.96 (5.72)	1.000	38.28 (5.96)	0.166	36.47 (4.51)	0.197
	RUP	38.36 (10.47)		34.27 (12.09)		38.40 (8.15)	
31.5 Nm	H	39.34 (7.35)	1.000	41.25 (7.73)	0.405	38.06 (8.62)	0.439
	RUP	40.13 (12.14)		36.74 (13.19)		40.71 (11.59)	
35 Nm	H	42.00 (6.94)	0.564	42.67 (5.55)	0.405	40.73 (6.34)	0.109
	RUP	42.00 (16.00)		39.18 (15.14)		42.70 (16.54)	

Abbreviations: H, unaffected tendon; MID, mid tendon; MTJ, musculotendinous junction; RUP, affected tendon; SWS, shear wave speed

Differences in shear wave speed were found at low level contractions (0, 3.5, and 7 Nm; *p* = 0.041, 0.006, and 0.023 respectively) between participants who experienced a MID lesion and participants who experienced a MTJ lesion. A significant difference between sides was found only at 3.5 and 7 Nm (*p* = 0.001 and *p* = 0.020) for the participants who had experienced a MID lesion. In the linear regression analysis conducted for each AT under different contraction intensities, the average coefficient of determination (R2) was 0.97 ± 0.02 for the unaffected side and 0.95 ± 0.04 for the affected side. This suggests a linear relationship between wave speed and ankle torque for both the affected and unaffected sides. No difference in the coefficient of determination was found (*p* = 0.174) between the affected and unaffected tendons.

### 3.3 Myotonometry

Stiffness analysis using myotonometry revealed a difference between the affected and the unaffected AT but only in the proximal part of the AT with a median (IQR) of 913 N/m (122) for the unaffected side and 825 N/m (115) for the affected side, showing decreased stiffness of the affected AT (*p* = 0.006).

### 3.4 Ultrasound Imaging

Ultrasound imaging revealed a significant difference in the thickness between the affected and the unaffected AT (*p* < 0.001) with a median (IQR) of 5.9 mm (1.6) for the unaffected and 10.1 mm (3.3) for the affected side.

CSA was different between sides in the distal and proximal part of the AT with a median (IQR) of 69.1 mm^2^ (17.9) and 115.0 mm^2^ (51.3) for the unaffected and affected side, respectively, in the distal part (*p* < 0.001) and 67.3 mm^2^ (26.3) and 135.0 mm^2^ (91.0) for the proximal part (*p* < 0.001).

## 4 Discussion

The main finding of this study evaluating AT loading using shear wave tensiometry in participants after ATR who had been treated conservatively is that there is no overall difference in shear wave speed values between the unaffected and the affected sides. At a long‐term follow‐up, the affected tendon restored loading capacity (measured with shear wave tensiometry) at a level that was comparable with that of the contralateral side. However, at low contraction levels, there was a difference in shear wave speed between tendons in the MID group.

Different behaviors of the AT at low contraction levels could depend on the tendon length. When subjected to a change in the ankle angle and to isometric contraction, the AT starts to increase its tension, and this onset of the rise in tension has been shown to be higher at lower strain values [28]. Affected tendons in the MID group could have a slightly different tendon length that leads to an increase in tension, visible particularly when contractions are of low intensity. Due to the limited sample size in the two subgroups, we cannot be certain that these findings will be replicated in a larger sample. However, they provide valuable preliminary insights that warrant further investigation.

High variability in tendon loading (large IQR values) has been shown in affected tendons compared to the unaffected side (Figure 3). This variability has also been observed in people with surgically treated ATR [20]. This could be related to the differences in muscle activation patterns between participants. Different plantar flexor muscles could contribute with different activation magnitudes to the net ankle torque, which is particularly true in patients after ATR where the flexor hallucis longus and the soleus have different contributions to the plantar flexion torque [13, 29, 30]. A different contribution of other plantar flexor muscles is probably emphasized more during low load contractions, although this different contribution cannot be measured with tensiometry located on the AT.

The variable behavior of tendons between participants during contractions was also evaluated using a linear regression between squared shear wave speed and ankle torque. In both tendons, the coefficient of determination R2 was close to 1 (0.97 and 0.95), showing a close relationship between shear wave speed and ankle torque. These results confirmed the consistency of the tensioned beam model principle [15] of the shear wave speed during contraction. No significant difference in the coefficient of determination (*p* = 0.174) was found between the affected and unaffected tendons, showing a similar linear behavior of the shear wave speed during contraction.

The present study showed similar shear wave tensiometry values overall between the affected and the unaffected sides, and these results are in line with other studies. Khair et al. [13] found no difference in AT stiffness between the affected and the contralateral sides 1 year after ATR, which was treated conservatively. Likewise, Bressel and McNair [31] found no difference in stiffness between the affected and contralateral sides when examined 1–5 years after ATR. Yoshida et al. [14] investigated ATR using shear wave elastography and showed that affected tendons (treated conservatively) reached values comparable to the unaffected side within the first 3 months after injury. However, this study showed that the stiffness values of the affected tendon exceeded that of the unaffected side 1 year after rupture. These differences could be explained by the different follow‐up periods between the studies and by the different technologies used in the detection of tendon mechanical properties. Indeed, shear wave elastography is a measure of tendon stiffness that uses sound waves and therefore is dependent on the tendon shear modulus, whereas in shear wave tensiometry, that evaluates the tendon under axial load, the shear modulus changes have negligible effects compared to the changes due to the axial stress [15]. These two technologies are not directly comparable as one measures the tension of the tendon under load, whereas the other assesses the elastic behavior of the tendon. Moreover, at present, shear wave elastography cannot evaluate the tendon under load [32, 33].

Other studies evaluating patients with ATR which have been treated operatively showed different long‐term mechanical properties of the tendon. In the initial stages of the post‐surgery healing phase (less than 1 year), the tendon appears to exhibit reduced stiffness [5, 6, 7, 8, 9] gradually progressing toward normal values or even surpassing those of the unaffected tendon in the later stages [10, 11, 20]. A previous study evaluating AT shear wave speed after ATR which was treated surgically showed a difference in tensiometry between the operated and unaffected AT at every level of contraction tested.

A possible explanation is that the suture performed during the surgery reacts differently to the loading compared to the natural scar formation following conservative treatment. As demonstrated in animal models, nonsurgical interventions result in a more advanced organization of collagen fibers, yielding a tendon with a healthier appearance compared to a higher proportion of scar tissues in those that received surgery [34]. To date, we cannot determine whether this phenomenon is also present in humans. While this may suggest that surgically treated tendons tend to exhibit higher shear wave speed values for a prolonged time after surgery, compared to conservatively treated tendons, these findings stem from separate studies. Therefore, it is not possible to directly compare the results, and caution should be exercised when interpreting these differences.

Finally, the different time points of analysis after rupture between the present study and the study analyzing surgically treated AT have to be taken into consideration [20]. In the study conducted in people following AT surgery [20], participants were evaluated 20.3 ± 8.1 months post‐surgery on average, which is earlier than those who underwent conservative treatment in the current study. We cannot exclude that the longer time elapsed may have led the affected tendon to return to values more similar to the contralateral side, although the correlations between shear wave speed and time after rupture were not significant in both the MID group (rho 0.155 *p* = 0.582) and MTJ group (rho 0.231 *p* = 0.427). Future studies that directly compare the two types of interventions should help to clarify possible tensiometry variations.

Unlike other technologies, such as shear wave elastography, shear wave tensiometry has the potential to measure tendon tension differences during loading. Combined with its precision in detecting changes in tendon status, this technology could enable a more reliable identification of subtle changes in tendon properties and, consequently, early detection of abnormalities.

At present, we cannot confirm whether increased or decreased tendon tension is associated with a higher risk of rupture or re‐rupture. In fact, only two studies [35, 36] reported that the contralateral tendon in patients who suffered an ATR exhibited reduced stiffness due to hypoxic degenerative tendinopathy, mucoid degeneration, or tendolipomatosis. The authors suggested that reduced stiffness in the contralateral tendon might indicate a higher risk of future rupture. Currently, we cannot assert that changes in tendon mechanical properties are a definitive risk factor for rupture or re‐rupture. Nevertheless, if future longitudinal studies confirm this association, having accurate technologies to assess tendon mechanical properties could become even more critical for identifying tendons at risk of rupture and implementing preventive strategies.

Myotonometry was used to evaluate the state of the tendon at rest. This, together with the assessment under load performed with shear wave tensiometry, enabled a better characterization of the AT behavior. Myotonometry evaluation of the AT revealed a significant difference of stiffness between the affected and the unaffected AT, but only in the proximal part of the AT with the results showing lower stiffness in the affected tendon (*p* = 0.006). These results appear to be in contradiction with shear wave tensiometry, which did not reveal any difference in tension between the tendons. Considering that these two technologies assess mechanical properties with different assumptions, a direct relationship between these two parameters might not be possible. To the best of our knowledge, this is the first study using myotonometry to assess people with ATR which has been treated conservatively. Other evaluations of patients with ATR which has been treated operatively reported increased stiffness [11, 20] of the operated tendon compared to the unaffected side. The different collagen compositions of scar tissue and the possible difference in tendon length after surgery can be factors that influenced the myotonometry results, leading to different values between tendons treated conservatively and those treated surgically.

Morphological properties of the tendon assessed via ultrasound imaging also revealed differences between the affected and unaffected tendons. Thickness and CSA were consistently higher in the affected tendon even beyond 1 year after ATR. These findings align with previous studies indicating a sustained increase in tendon thickness and CSA in the later stages after surgery [6, 7, 11, 13, 20] and even after conservative treatment [13, 14].

Ruptured tendons exhibit an irregular, rounded region of enlarged size, characterized by disorganized collagen fibers resulting from scar maturation and remodeling [37]. Ultrasound images were acquired at 2–7 cm from the calcaneal insertion and this is the region where the ATR mostly occurs [38]; therefore, this is where a large collagen accumulation and scar tissue formation are expected, leading to an increase tendon size. Despite the morphological changes observed in this study (increased thickness and CSA) that can last many years after ATR, the mechanical properties seem to remain similar to the unaffected side.

Patient‐reported outcomes were in line with previous literature [39, 40], showing a low level of disability post‐ATR. The mean score on the ATRS was 86/100 with 72% of participants returning to a level of activity similar to the level before the rupture. Comparing across studies, the current cohort of patients who had ATR treated conservatively showed similar functional disability, measured with the ATRS score, to those treated surgically 1 year after the rupture [20]. These results are also supported by a recent clinical trial that showed similar outcomes between the two types of treatment [3]. However, despite good ATRS scores, this study revealed persistent differences in strength and muscle trophism of the triceps surae. The calf on the affected side remains weaker (lower MVC, *p* < 0.001) and with a smaller circumference (*p* < 0.001) in the longer term after ATR. These results are comparable with a previous study [40] that showed a reduced torque of the plantar flexor muscles long after ATR.

### 4.1 Study Limitations

There are some limitations of this study that need to be acknowledged. First, the post rupture rehabilitation protocol was not recorded; therefore, it was not possible to establish whether the measured outcomes were influenced by differences in rehabilitation protocols. Second, a different load distribution between the two tendons might be associated with the different resting lengths of the tendon following rupture. The length of an affected AT could have been modified by different factors and was not measured in this study. The position of the foot in the cast and the potential lengthening during the rehabilitation phase were elements that could have contributed to a different tendon length. We could therefore not establish if the tendon resting length influences shear wave speed values. Lastly, even if no correlation was found between shear wave speed and time elapsed since the rupture, we cannot exclude that the mechanical properties of the affected tendon could have changed also in relation to the different time points of the evaluation. Additional research is required to gain a more comprehensive understanding of the clinical significance of shear wave tensiometry in assessing tendon loading and informing the management of ATR.

## 5 Conclusion

Shear wave tensiometry can quantify tendon shear wave speed after ATR which has been treated conservatively. Overall, there is no difference in shear wave speed values between the affected and the unaffected AT when measured in the longer‐term follow‐up after ATR. However, some differences were detected in shear wave speed between MID and MTJ lesions at low contraction intensities, with side‐to‐side differences in participants who had experienced a MID lesion. Moreover, tendon thickness and cross‐sectional area, as well as plantar flexor strength, remain different between the affected and the unaffected AT.

## Author Contributions

**Alessandro Schneebeli:** conceptualization, data curation, formal analysis, investigation, writing – original draft, writing – review and editing. **Giuseppe Filardo:** conceptualization, supervision, writing – original draft, writing – review and editing. **Enrique Testa:** conceptualization, writing – original draft, writing – review and editing. **Martin Riegger:** conceptualization, writing – original draft, writing – review and editing. **Alessandro Sangiorgio:** formal analysis, investigation, writing – original draft, writing – review and editing. **Deborah Falla:** conceptualization, supervision, writing – original draft, writing – review and editing. **Corrado Cescon:** data curation, investigation, software, writing – original draft, writing – review and editing. **Emiliano Soldini:** methodology, writing – original draft, writing – review and editing. **Marco Barbero:** conceptualization, project administration, supervision, writing – original draft, writing – review and editing.

## Ethics Statement

Ethical approval for this study was obtained from the Ethics Committee of Canton Ticino (ID:2022‐01707/CE4192).

## Consent

All the included participants provided written informed consent.

## Conflicts of Interest

The authors declare no conflicts of interest.

## Data Availability

The dataset used and/or analyzed is available from the corresponding author on request.
